# Analysis of MAT3 gene expression in NSCLC

**DOI:** 10.1186/1746-1596-8-166

**Published:** 2013-10-09

**Authors:** Shangen Zheng, Yuwen Du, Heying Chu, Xudong Chen, Ping Li, Yuanyuan Wang, Yunyun Ma, Huaqi Wang, Wenqiao Zang, Guojun Zhang, Guoqiang Zhao

**Affiliations:** 1 Department of Blood Transfusion, Wuhan General Hospital of Guangzhou Military Command, Wuhan 430070, China; 2 College of Basic Medical Sciences, Zhengzhou University, No.100 Kexue Road, Zhengzhou 450001, China; 3 Department of Respiratory Medicine, the First Affiliated Hospital of Zhengzhou University, Zhengzhou 450052, China; 4 Department of Histology and Embryology, Luohe Medical College, Luohe 462002, China

**Keywords:** Non-small-cell lung cancers (NSCLC), MAT3, Gene expression

## Abstract

**Background:**

Many studies have suggested different roles of Metastasis-associated protein 3 (MAT3) in different types of human cancers. However, expression of MAT3 in primary lung cancer and its relationship with clinicopathological factors have not been examined and the biological roles of MTA3 in lung cancer cells are still unclear.

**Methods:**

The expression of MAT3 mRNA and protein were detected with quantitative real-time RT-PCR and immunohistochemical methods in 118 NSCLC samples and corresponding non-neoplastic samples. Survival curves were made with follow-up data. The relations of the prognosis with clinical and pathological characteristics were analyzed.

**Results:**

The expression level of MAT3 mRNA and the positive rate of MAT3 protein were significantly higher in NSCLC samples than that in non-neoplastic samples, and in NSCLC samples with lymph node metastasis than that in NSCLC samples without lymph node metastasis (P < 0.01). MAT3 mRNA expression level was a risk factor of lymph node metastasis in patients with NSCLC (P = 0.006). There were significant differences in survival curves between lymph node metastatic group and non-metastatic group (P = 0.000), among groups of MAT3 positive and negative (P = 0.000), among groups of TNM stage I, II and III (P = 0.000) and among groups of tumor status T1, T2 and T3T4 (P = 0.000); but no statistical significance between male patients and female patients (P = 0.516), between ≥60 years old patients and <60 years old patients (P = 0.133), between histology types adenocarcinoma and squamous cell carcinoma (P = 0.865) and between well differentiation and moderate-poor differentiation (P = 0.134). The level of MAT3 mRNA (P = 0.000) and protein (P = 0.000) were risk factors of survival.

**Conclusion:**

Our study showed that MAT3 over-expression in NSCLC tissue, and MAT3 mRNA level is a risk factor of lymph node metastasis. The level of MAT3 mRNA and protein were risk factors of survival in patients with NSCLC. It suggested that this antigen could be used as a simple and efficient parameter with which to identify high-risk patients.

**Virtual slides:**

The virtual slides for this article can be found here: http://www.diagnosticpathology.diagnomx.eu/vs/5585901065503943.

## Introduction

Metastasis-associated protein 3 (MTA3) was originally found as a member of a small protein family (including MTA1, MTA2 and MTA3), which is a constituent of the Mi-2/nucleosome remodeling and deacetylase (NuRD) protein complex that regulates gene expression by altering chromatin structure and can facilitate cohesin loading onto DNA [1-8].

MTA3 was reported to participate in B lymphocyte development, in plasmacytoma cell lines, the overexpression of BCL6 (B-cell CLL/lymphoma 6) and MTA3 downregulated plasma cell differentiation genes [9]. Since then, however, the expression of MTA3 has been found reduced in breast cancer, endometrial cancer and ovarian cancer [10-12]. MTA3 upregulation prevents epithelial-mesenchymal transition (EMT) by directly repressing Snail expression, thereby upregulating E-cadherin protein levels in breast cancer [8,13]. Moreover, MTA3 was reported as an independent and unfavorable prognostic marker in uterine non-endometriod carcinoma [14]. These findings suggest that the expression of MTA3 is closely related to invasiveness, metastasis and prognosis of tumour.

Lung cancer is one of the leading causes of all cancer-related deaths worldwide and its incidence is increasing [15,16]. The majority of diagnosed lung cancer cases are non-small-cell lung cancers (NSCLCs). Many previous studies showed the expression and functions of proteins, genes and enzymes in lung cancer [8,17,18]. However, expression of MTA3 mRNA and protein in primary lung cancer and its relationship with clinicopathological factors has not been examined and the biological roles of MTA3 in lung cancer cells are still unclear.

To explore the role of MTA3 in NSCLC, we analysed 118 cases of NSCLC patients retrospectively between 2001 and 2005, detected the expression of MTA3 mRNA and protein with real time RT-PCR and immunohistochemical methods, and explore the relationship between expression of MTA3 mRNA and protein and survival time.

## Materials and methods

### Clinical sample collection

Between 1.1.2001 and 31.7.2005, 118 patients with NSCLC were enrolled in this study from Wuhan General Hospital of Guangzhou Military and the First Affiliated Hospital of Zhengzhou University. Patients who had recurrent NSCLC or primary NSCLC but received chemoradiotherapy before surgical operation were excluded. Of the 118 patients, 59 were female and 59 were male. And there were 49 cases with lymph node metastasis, 69 cases without lymph node metastasis. We obtained paired NSCLC and adjacent non-tumor lung tissues (located more than 5 cm away from the tumors) from 118 patients who underwent primary surgical resection of NSCLC with informed consent. Both tumor and non-tumor samples were confirmed as such by pathological examinations. These samples were snap-frozen in liquid nitrogen after resection. This study was approved by the ethics committee of Zhengzhou University and informed consent was obtained from each patient (Table 1).

**Table 1:** Clinicopathologic characteristics of the 118 NSCLC cases

**Parameter**	**Category**	**n**
Gender	Male	59
	Female	59
Age (years)	≥60	66
	<60	52
Histology	Adeno	69
	SCC	49
Differentiation	Well	46
	Moderate -poor	72
Tumor stage	T1	39
	T2	55
	T3–4	24
TNM stage	I	52
	II	42
	III	24
Node status	Positive	49
	Negative	69

### Immunohistochemistry

The MTA3 protein was detected by a rabbit polyclonal antibody (Santa Cruz USA). The sections were stained with streptavidin peroxidase (SP) kit (Maixin Biotechnology Company, China), visualized with DAB coloration kit (Boaosen Company, China), followed by counterstaining of campeachy, dehydration, transparency and mounting [17]. All slides were evaluated by two different pathologists and then in conference in a blinded manner without any prior knowledge of the clinicopathological parameters. Negative controls of immunohistochemical reactions were performed by omitting the primary antibody. Replacement of primary antibody by PBS was used as blank control. Staining intensity was modified. Briefly, gray-scale digitized images were imported into the Optimas software (Optimas 6.0, Optimus Corp., Bothell, WA, USA). Control staining (without primary antibody) was used for the extraction of the background staining. In every case, control reactions were included, in which specific antibody was substituted by the Primary Mouse Negative Control.

### Real-time fluorescent quantitative RT-PCR

MTA3 and GAPDH primers were designed according to MTA3 mRNA (NM_020744) and GAPDH mRNA (NM_002046) with Oligo 6.0 software. The sequences were as follows: MTA3 sense 5′ TATCAGGGGAAAGTGCAGTGTTG 3′, MTA3 antisense 5′-AACAGCATTTCTGGAATGTCTGC-3′, GAPDH sense 5′-GCAAATT CCATGGCACCGTCAAG-3′, GAPDH antisense 5′-GTGGTGAAGACGCCAG TGGACTC-3′. The length of fragments amplified by PCR with MTA3 primers and GAPDH primers were 183 bp and 151 bp, respectively. All the primers were synthesized by Sangon Biotech (Shanghai) Co., Ltd. RNAs were extracted from 118 NSCLC samples with RNA extraction kit (Qiagen) and then cDNAs were synthetized by AMV (Promega). Aliquots of the reaction mixture were used for the qPCR amplification with the IQ5 system (Bio-Rad) using IQ SYBR Green Supermix (Bio-Rad). The PCR was run for 40 cycles of denaturation at 95°C for 15 sec, annealing at 55°C for 15 sec and elongation at 72°C for 15 sec. Gene expression was quantified by the comparative CT method, by normalizing CT values to the housekeeping gene GAPDH. After amplification, melting curve analysis was performed to ensure the specificity of the products.

### Statistical analysis

Statistical analysis was performed using SPSS17.0 software. Data were expressed as means ± standard deviation (SD). Student’s *t* test was used in the comparison of mean between two samples. Fourfold table Chi square test was used in the comparison of ratios between two samples. Logistic analysis was used in the correlation of lymph node metastasis with MAT3 mRNA expression. The follow-up data was analyzed by the Kaplan-Meier method and log-rank test. Cox proportional hazards model were used in multivariate prognostic analysis. P values less than 0.05 were considered statistically significant.

## Results

### MTA3 mRNA is over-expressed in NSCLC samples and is a risk factor of lymph node metastasis

MTA3 mRNA is over-expressed in NSCLC samples and is a risk factor of lymph node metastasis. The relative expression level of MTA3 mRNA was significantly higher in NSCLC samples (118 samples, 0.2494 ±0.10361) than that in non-neoplastic samples(118 corresponding samples, 0.1578 ± 0.07694) (P = 0.002), and in NSCLC samples with lymph node metastasis(49 samples, 0.2810 ± 0.08593) than that in NSCLC samples without lymph node metastasis (69 samples, 0.2270 ± 0.10969) (P = 0.003) (Table 1). Logistic regression analysis indicated that the relative expression level of MTA3 mRNA was a risk factor of lymph node metastasis in the patients with NSCLC (Wald *χ*^2^ = 7.493, P = 0.006).

### Positive rate of MTA3 protein is high in NSCLC samples and associated with lymph node metastasis

**Figure 1:**
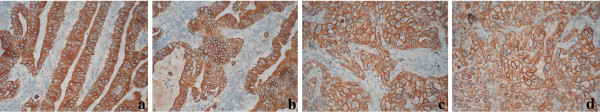
**Immunohistochemical staining of MTA3 in lung cancer tissue sections. a** and **b**: Positive MTA3 staining in a case of lung adenocarcinoma. **c** and **d**: Positive MTA3 staining in a case of squamous cell carcinoma.

MTA3 showed positive immuno-reactivity mainly in nucleus and cytoplasms of the cells (Figure 1). The positive rate of MTA3 protein expression was significantly higher in NSCLC samples (59.32%, 70/118) than that in non-neoplastic samples (0.00%, 0/118), and in NSCLC samples with lymph node metastasis (79.59%, 39/49) was higher than that in NSCLC samples without lymph node metastasis (44.93%, 31/69) (P =0.000). Statistical differences were found between different ages (P = 0.038), differentiation (P = 0.001), tumor stage (P = 0.018) and TNM stage (P = 0.018) (Table 2). There were no statistical differences between different genders (P = 1.000) and histology (P = 0.849) (Table 2).

**Table 2:** Expression of MTA3 mRNA and protein in the 118 NSCLC cases

**Parameter**	**MTA3 mRNA**		**MTA3 protein**		
	**Expression level**	**P value**	**n**		**P value**
			**Negative**	**Positive**	
Gender					
Male	0.2547 ± 0.10485	0.581	24	35	1.000
Female	0.2442 ± 0.10298		24	35	
Age (years)					
≥60	0.2646 ± 0.09439	0.073	21	45	0.038*
<60	0.2302 ± 0.11224		27	25	
Histology					
Adeno	0.2444 ± 0.10711	0.534	29	40	0.849
SCC	0.2565 ± 0.09913		19	30	
Differentiation					
Well	0.2234 ± 0.11671	0.038*	28	18	0.001*
Moderate-poor	0.2661 ± 0.09131		20	52	
Tumor stage					
T1	0.2233 ± 0.10521		22	17	0.018*
T2	0.2485 ± 0.10680	0.030*	21	34	
T3–4	0.2942 ± 0.07934		5	19	
TNM stage					
I	0.2350 ± 0.10463	0.079	28	24	0.018*
II	0.2435 ± 0.10970		15	27	
III	0.2913 ± 0.08077		5	19	
Nodal status					
Positive	0.2810 ± 0.08593	0.003*	10	39	0.000*
Negative	0.2270 ± 0.10969		38	31	

*Indicated statistical significance (*P* < 0.05).

### Expression level of MTA3 mRNA and protein are risk factors of survival in patients with NSCLC

**Figure 2:**
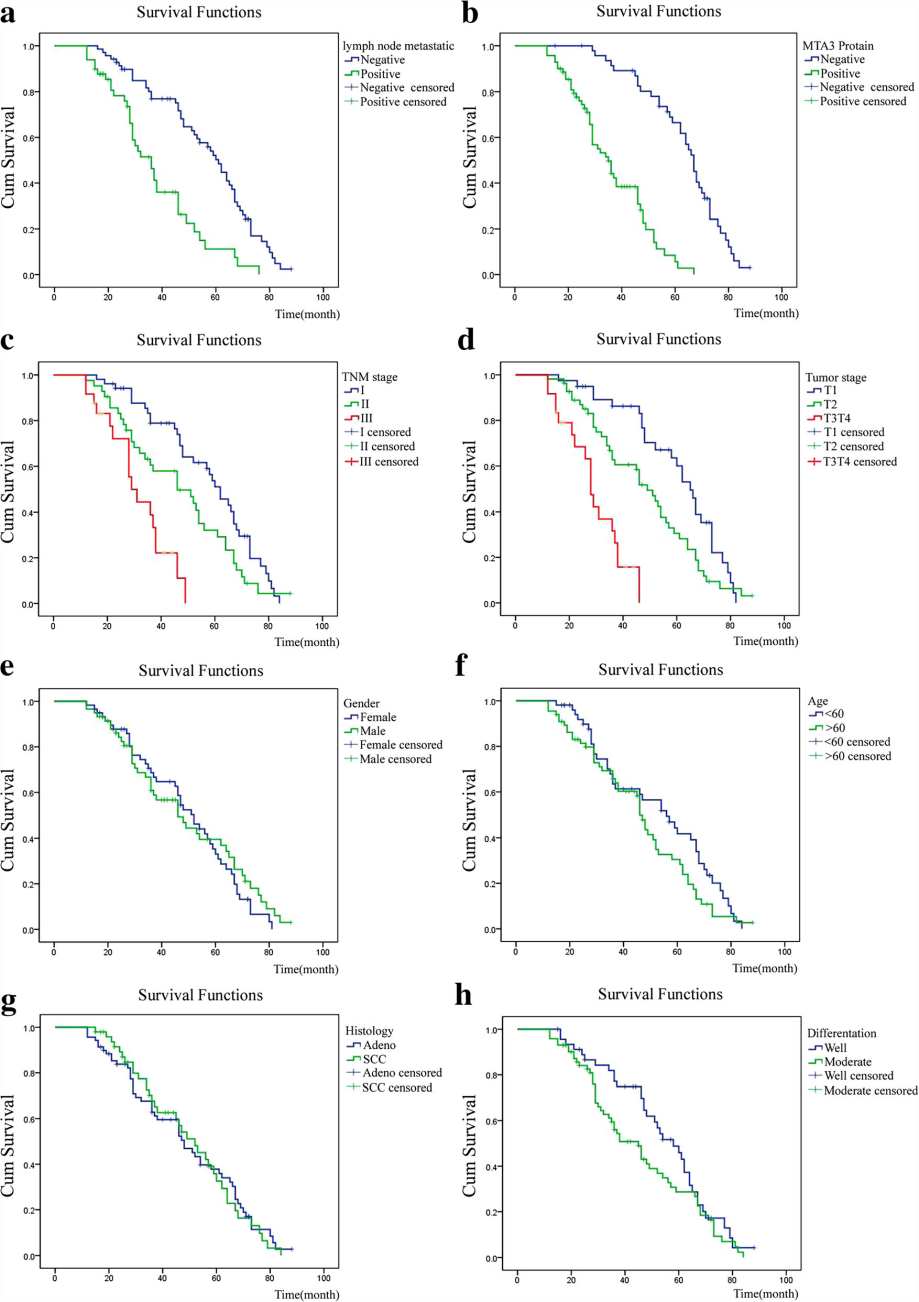
**Kaplan–Meier curves of the clinical outcome for MTA3 regarding MTA3 immunolabelling. a**: Schematic representation shows survival curves of lymph node metastatic group and non-metastatic group in patients with NSCLC (P = 0.000). **b**: Schematic representation shows survival curves of different MTA3 protein level groups in patients with NSCLC (P = 0.000). **c**: Schematic representation shows survival curves of different TNM stages in patients with NSCLC (P = 0.000). **d**: Schematic representation shows survival curves of different tumor stages in patients with NSCLC (P = 0.000). **e**: Schematic representation shows survival curves of different genders in patients with NSCLC (P = 0.516). **f**: Schematic representation shows survival curves of different ages in patients with NSCLC (P = 0.133). **g**: Schematic representation shows survival curves of different histology types in patients with NSCLC (P = 0.865). **h**: Schematic representation shows survival curves of differentiations in patients with NSCLC (P = 0.134).

Survival curves were drawn using SPSS17.0 software with Kaplan-Meier method. Log-rank test indicated that there were significant differences in survival curves between lymph node metastatic group and non-metastatic group (*χ*^2^ =22.810, P = 0.000, Figure 2a), among groups of MTA3 protein expression positive and negative (*χ*^2^ = 52.161, P = 0.000, Figure 2b), among groups of TNM stage I, II and III (*χ*^2^ = 27.037, P = 0.000, Figure 2c) and among groups of tumor status T1, T2 and T3T4 (*χ*^2^ = 37.585, P = 0.000, Figure 2d); but no significant differences between male patients and female patients (*χ*^2^ = 0.423, P = 0.516, Figure 2e), between ≥60 years old patients and <60 years old patients (*χ*^2^ = 2.261, P = 0.133, Figure 2f), between histology types (adeno and SCC) (*χ*^2^ = 0.029, P = 0.865, Figure 2g) and between well differentiation and moderate-poor differentiation (*χ*^2^ = 2.249, P = 0.134, Figure 2h).

## Discussion

MTA3 is the latest addition to the MTA family. It was identified as an estrogen-dependent component of the Mi-2/NuRD transcriptional corepressor in breast epithelial cells [8]. Through a MTA3-NuRD complex, MTA3 represses Wnt4 transcription and Wnt4 secretion, inhibiting Wnt-target genes in mammary epithelial cells [18,19]. The absence of MTA3 as well as the absence of estrogen receptor (ER) results in an aberrantly increased expression of the transcriptional repressor Snail, a master regulator of EMT [20-22]. It has also been reported that expression of MTA3 inhibits ductal branching in virgin and pregnant mammary glands in MTA3-transgenic mice [23], where the inappropriate development of mammary glands results in the development of hyperplastic nodules and mammary tumors, including adenocarcinomas and lymphomas [24-26]. Considering the many reports showing the clinical relevance of the expression of MTA3, it is likely that MTA3 represents master co-regulatory molecules involved in the carcinogenesis and progression of various malignant tumors.

This study confirmed the presence of MAT3 overexpression in NSCLC samples for the first time. The expression level of MAT3 mRNA and the positive rate of MAT3 protein expression were significantly higher in NSCLC samples (0.2494 ±0.10361 and 59.32%) than that in non-neoplastic samples (0.1578 ± 0.07694 and 0.00%), and in NSCLC samples with lymph node metastasis (49 samples, 0.2810 ± 0.08593) than that in NSCLC samples without lymph node metastasis (69 samples, 0.2270 ± 0.10969) (all P < 0.01). The expression level of MAT3 mRNA was positively correlated with lymph node metastasis, and was a risk factor of lymph node metastasis in the patients with NSCLC (Wald *χ*^2^ = 7.493, P = 0.006). There were significant differences in survival curves between lymph node metastatic group and non-metastatic group (*χ*^2^ =22.810, P = 0.000), among groups of MAT3 protein expression positive and negative (*χ*^2^ = 52.161, P = 0.000), among groups of TNM stage I, II and III (*χ*^2^ = 27.037, P = 0.000) and among groups of tumor status T1, T2 and T3T4 (*χ*^2^ = 37.585, P = 0.000); but no significant differences between male patients and female patients (*χ*^2^ = 0.423, P = 0.516), between ≥60 years old patients and <60 years old patients (*χ*^2^ = 2.261, P = 0.133), between histology types (adeno and SCC) (*χ*^2^ = 0.029, P = 0.865) and between well differentiation and moderate-poor differentiation (*χ*^2^ = 2.249, P = 0.134). The level of MAT3 mRNA and protein were risk factors of survival, but lymph node metastasis was not a risk factor of survival in the patients with NSCLC. These results suggest that there is MAT3 over-expression in NSCLC samples and the level of MAT3 mRNA is a risk factor of lymph node metastasis and survival in the patients with NSCLC. The results may provide a basis for exploring the role of MAT3 in NSCLC. The expression level of MAT3 is expected to become an important index to assess NSCLC invasion and metastasis, and MTA3 may be a useful marker to assess and identify high-risk patients with NSCLC.

## Abbreviations

NSCLC: Non-small-cell lung cancers; SCC: Squamous cell carcinoma; MTA3: Metastasis-associated protein 3; BCL6: B-cell CLL/lymphoma 6; EMT: Epithelial-mesenchymal transition; ER: Estrogen receptor.

## Competing interests

The authors declare that they have no competing interests.

## Authors’ contributions

GQZ, GJZ and SGZ: conceived of the study, and participated in its design and coordination and helped to draft the manuscript. SGZ, YWD, XDC, HYC and HQW: collected the samples. SGZ, YWD, PL, YYW, YYM and WQZ: carried out part of experiments and wrote the manuscript. YYW, GJZ and GQZ performed the statistical analysis. All authors read and approved the final manuscript.
